# Eales disease: no typical clinical presentation

**DOI:** 10.11604/pamj.2014.17.136.4019

**Published:** 2014-02-26

**Authors:** Belmokhtar Adil, Daoudi Rajaa

**Affiliations:** 1 University Mohamed V Souissi, Hôpital des Spécialités, Ophtalmologie A Department, Morocco

**Keywords:** Eales disease, retinal phlebitis, visual acuity

## Image in medicine

Eales disease is clinically manifested by retinal phlebitis, the onset of ischemia and retinal vessels neo area is often revealed by vitreous hemorrhage. Its etiology remains unknown. The management of this disease depends on the clinical presentation. Our patient was 27 years old, consults for a progressive loss of visual acuity unilateral in her left eye. On fundus exam at the emergency department, we found a vitreous hemorrhage, retinal hemorrhage, periphlebitis and retinal periphery ischemia (A). Laboratory tests were negative for potentially occlusive vasculopathy. Angiogram shows retinal neovascularization (B) and peripheral ischemic lesions (C, D). The patients received treatment with corticosteroids supplemented by laser photocoagulation. Improved visual acuity was obtained.

**Figure 1:**
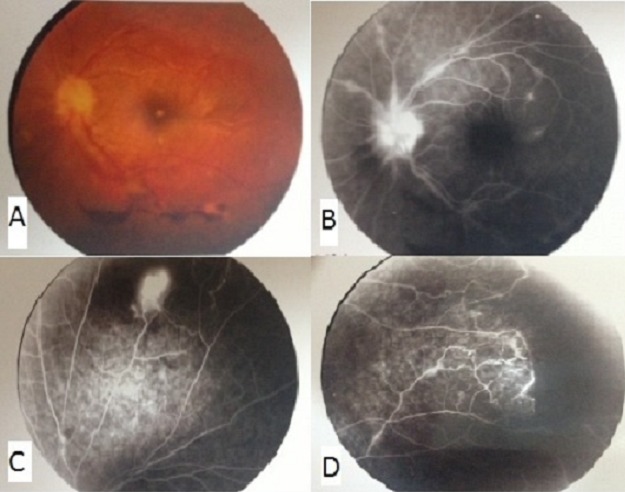
A) vitreous hemorrhage, retinal hemorrhage, periphlebitis and retinal periphery ischemia; (B, C, D): shows retinal neovascularization and peripheral ischemic lesions

